# Distinctive T-cell receptor repertoire in paediatric inflammatory multisystem syndrome temporally associated with coronavirus disease 2019/multisystem inflammatory syndrome in children patients: possible thymus involvement

**DOI:** 10.1093/cei/uxaf027

**Published:** 2025-05-04

**Authors:** Diana C Yanez, Jasmine Rowell, Maximillian Woodall, Stuart Adams, Lauran O’Neill, Konstantinos Mengrelis, Ching-In Lau, Susan Ross, Sarah Benkenstein, Kate Plant, Claire M Smith, Benny Chain, Mark J Peters, Tessa Crompton

**Affiliations:** Infection, Immunity and Inflammation Research & Teaching Department, UCL Great Ormond Street Institute of Child Health, London, UK; Infection, Immunity and Inflammation Research & Teaching Department, UCL Great Ormond Street Institute of Child Health, London, UK; Infection, Immunity and Inflammation Research & Teaching Department, UCL Great Ormond Street Institute of Child Health, London, UK; Great Ormond Street Hospital, London, UK; Great Ormond Street Hospital, London, UK; Infection, Immunity and Inflammation Research & Teaching Department, UCL Great Ormond Street Institute of Child Health, London, UK; Infection, Immunity and Inflammation Research & Teaching Department, UCL Great Ormond Street Institute of Child Health, London, UK; Infection, Immunity and Inflammation Research & Teaching Department, UCL Great Ormond Street Institute of Child Health, London, UK; Great Ormond Street Hospital, London, UK; Great Ormond Street Hospital, London, UK; Infection, Immunity and Inflammation Research & Teaching Department, UCL Great Ormond Street Institute of Child Health, London, UK; Division of Infection and Immunity, University College London, London, UK; Infection, Immunity and Inflammation Research & Teaching Department, UCL Great Ormond Street Institute of Child Health, London, UK; Great Ormond Street Hospital, London, UK; Infection, Immunity and Inflammation Research & Teaching Department, UCL Great Ormond Street Institute of Child Health, London, UK

**Keywords:** PIMS-TS, MIS-C, COVID-19, thymus, TCR repertoire, TREC

## Abstract

During the coronavirus disease 2019 (COVID-19) pandemic a rare new paediatric inflammatory condition (paediatric inflammatory multisystem syndrome temporally associated with COVID-19 (PIMS-TS)/MIS-C) was identified which correlated with previous or recent SARS-CoV-2 infection. PIMS-TS led to severe multiorgan inflammation, suggestive of disruption of central tolerance and thymus function. Here we investigated the possible role of the thymus in paediatric PIMS-TS. We confirmed that human thymus explants can be infected with SARS-CoV-2 in vitro. Comparison of T-cell populations in blood from PIMS-TS patients and age-matched healthy control children showed that although the overall proportions of CD4 and CD8 T-cell populations were decreased in PIMS-TS patients, the proportion of naïve cells in the CD4 population was higher in the PIMS-TS group. In PIMS-TS patients, the number of TREC in Peripheral blood mononuclear cells (PBMC) correlated strongly with the proportion of naïve CD4 and CD8 T cells, whereas this correlation was not present in healthy children. Sequencing rearranged TCRβ and TCRɑ transcripts from FACS-sorted CD4+CD8-CD3+ and CD4-CD8+CD3+ from blood from PIMS-TS, healthy children, and additionally paediatric severe COVID-19 patients showed that while all three groups showed similar diversity and distribution, the repertoire of the PIMS-TS and COVID-19 groups had distinctive patterns of TCR gene segment usage and VJ combinatorial usage compared to healthy controls (TRBV11-2 × TRBJ2-7, TRBV11-2 × TRBJ1-1, TRBV11-2 × TRBJ2-5, TRBV11-2 × TRBJ2-1; TRBV29-1 × TRBJ2-7, TRBV29-1 × TRBJ1-1 enriched in PIMS-TS; TRBV7-9 × TRBJ1-2, TRAV9-2 × TRAJ30, and TRAV26-1 × TRAJ39 enriched in COVID-19). The non-productive TCR rearrangements in the PIMS-TS group were also enriched for TRBV11-2, and showed bias towards distal (5′TRAV to 3′TRAJ) TCRɑ gene segment usage, suggesting involvement of the thymus in PIMS-TS.

## Teaser

Our study has identified distinctive characteristics of the TCR repertoires of children hospitalized with PIMS-TS. We found several similar features to previously described repertoires from PIMS-TS 2020 cohorts, as well as novel features of their TCR repertoires. Although our analyses do not definitively demonstrate thymus involvement in PIMS-TS, several aspects of these TCR repertoires are suggestive of thymus dysfunction, including changes in proportional combinatorial V × J usage in productive and non-productive TCR sequences with bias to 5′TRAV × 3′TRAJ rearrangements.

## Graphical Abstract

**Graphical Abstract:**
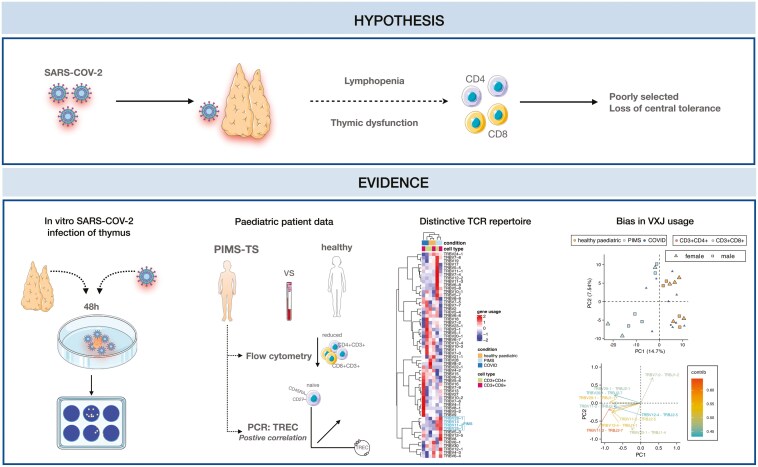

## Introduction

The thymus is essential for T-cell development and TCR repertoire selection but undergoes changes in output and function across the life course [1, 2]. Thymus size and output are greatest during infancy, but decline in adult life [3]. This thymus atrophy is an evolutionarily conserved process which begins early in life [4], and the thymus progressively decreases in size but continues to produce T cells and replenish the peripheral T-cell pool into middle age [5]. Investigation of immunity in patients who have undergone thymectomy have demonstrated the importance of thymus function across the life course [5–10]. In old age, thymus function deteriorates leading to reduced T-cell output and quality, compromising the replacement of T cells in the periphery and causing poorer immunity to viral infection and increased inflammation [11].

In the thymus signals provided by thymic epithelial cells (TEC) support the development of T cells and determine the T-cell receptor (TCR) repertoire: cortical TEC provide MHC + peptide ligands for positive selection, and medullary (m) TEC induce tolerance by providing MHC + peptide ligands to drive regulatory T-cell (Treg) maturation and clonal deletion of self-reactive clones [12]. Mature mTEC express AIRE which enables the expression of Tissue Restricted Antigens (TRA) to induce self-tolerance. AIRE mutation leads to multiorgan autoimmunity, highlighting the importance of the thymus for self-tolerance and immune regulation [12].

During T-cell development, the T-cell Receptor Beta locus (*TRB)* and T-cell Receptor Alpha locus (*TRA*) are sequentially rearranged to produce T cells with a diverse TCR repertoire [13]. Allelic exclusion of the *TRB* locus prevents developing thymocytes from simultaneously rearranging and expressing two TCRβ chains, but the *TRA* locus simultaneously undergoes biallelic V-J rearrangement [14–16]. The *TRA* locus can undergo multiple rounds of rearrangement on each chromosome, sequentially moving 3′ to 5′ along the series of TRAV gene segments, while moving 5′ to 3′ along the series of TRAJ segments, with proximal pairs (3′ TRAV segments with 5′ TRAJ segments) initiating this sequence of rearrangements and distal pairs (5′TRAV to 3′ TRAJ rearrangements) taking place later in the cell after initiation of *TRA* rearrangement [14, 15, 17]. Thus, in situations in which the thymus is recovering quickly from depletion, such as after hydrocortisone treatment, proximal 3′TRAV to 5′TRAJ rearrangements are more frequent [18].

Coronavirus disease 2019 (COVID-19) disproportionately affects the elderly, and in severe disease, peripheral T cells are depleted and exhausted, whereas dysregulated immune responses and inflammation are prevalent. T-cell responses are required to control and eliminate SARS-CoV-2 infection, but severe COVID-19 is associated with lymphopenia and impaired T-cell responses [19–22]. Thymus function should therefore be important in COVID-19 patients to replenish peripheral T-cell populations so differences in thymus function could be one factor that accounts for the difference in survival between young and old: very few children die from COVID-19, and children quickly recover their peripheral T-cell pool after depletion.

Early during the COVID-19 pandemic, a rare new paediatric inflammatory condition dubbed paediatric inflammatory multisystem syndrome temporally associated with COVID-19 (PIMS-TS; also known as a multisystem inflammatory syndrome in children (MIS-C)) was identified which correlated with previous/recent SARS-CoV-2 infection [23–25]. PIMS-TS shared clinical features with Toxic Shock Syndrome and Kawasaki disease and involved severe multiorgan inflammation, associated with fever, shock, skin, gastrointestinal and cardiac disorders [26, 27]. PIMS-TS typically arose between 2 and 6 weeks after SARS-CoV-2 infection, which may have been asymptomatic or very mild at the time. It was unclear how the virus led to this disorder and why it particularly affected children. One theory has linked PIMS-TS to monogenic autosomal recessive deficiencies in *OAS1*, *OAS2,* and *RNASEL*, leading to heightened inflammatory cytokine response, while several studies suggested that PIMS-TS is caused by clonal expansion of T cells bearing TCRs reactive with a superantigen-like epitope within the SARS-CoV-2 spike protein leading to disease akin to Toxic Shock Syndrome [28–33]. However, a recent study that experimentally investigated the direct interaction between TCR and the spike protein by surface plasmon resonance found no interaction, indicating that it is very unlikely that the spike protein acts as a superantigen [34].

The pattern of broad multisystem inflammation observed in PIMS-TS is suggestive of possible disruption of central tolerance and thymus function. We, therefore, hypothesized that while active thymus function might provide children with an advantage in their recovery from COVID-19, in the case of PIMS-TS, thymus dysfunction following SARS-CoV-2 infection of the thymus might drive disease some weeks after the initial infection, as inappropriately selected T cells are released into the peripheral T-cell pool. Under this scenario, the increased thymus output in children would provide a disadvantage in the period during which the thymus recovered from SARS-CoV-2 infection in comparison to adults, as more inappropriately selected T cells would be rapidly released into the periphery. In support of a possible role for the thymus in PIMS-TS, a recent study has shown that TEC can be infected with SARS-CoV-2 in severe COVID-19, affecting thymus function [35].

To investigate the potential role of the thymus in paediatric PIMS-TS, we therefore analysed blood T-cell populations from PIMS-TS patients compared to that of healthy control children. We assess the hypothesis that PIMS-TS is caused by rare SARS-CoV-2 infection of the thymus, which then affects the repertoire, function, and immune regulation of recently replenished peripheral T-cell populations.

## Materials and methods

### Patient samples

Human thymus tissue was obtained from thymus removed from infants during elective cardiac surgery at Great Ormond Street Hospital (GOSH), London, UK, with informed consent from parents, under ethical approval from the UK Health Research Authority (HRA).

Blood samples (3–5 ml venous whole blood) were obtained from patients on their first day of hospitalization in the Paediatric Intensive Care Unit (PICU) of GOSH between March 2021 and March 2022. All PIMS-TS patients (*n* = 6, 10–16 years, 4 males and 2 females) were SARS-CoV-2 IgG+, and previously fit and well. COVID-19 patients (*n* = 6, 10–16 years, 4 females, 2 males; *n* = 3, <5 years, all females) were PCR + on admission and several had underlying health conditions. Further patient details are provided in Table 1. The study was carried out in compliance with good clinical practice and under ethical approval from the UK HRA research ethics committee. Parents provided informed written consent. Peripheral blood mononuclear cells (PBMC) were isolated from whole blood by Lymphoprep (StemCell Technologies) density gradient centrifugation. Some PBMC were used for FACS staining and TREC analysis (Fig. 2), with healthy controls as described in [10]. The remainder were aliquoted in freezing medium (10% DMSO in FBS) and cryopreserved in liquid N_2_ for later analysis of TCR repertoire, with frozen PBMC from healthy paediatric donors (*n* = 5; age 10–16 years, 3 females, 2 males for CD8 populations, 2 females and 2 males for CD4 populations) obtained in accordance with their guidelines, from the Biobank of the Centre for Adolescent Rheumatology Versus Arthritis at UCL, UCLH, and GOSH, which received ethical approval from HRA, reference: REC11/LO/0330 (London-Harrow Research Ethics Committee. Healthy control blood samples were archived before 2020.

**Table 1:** Patient information

	Sex	Age	Diagnosis	Additional diagnosis	Test results on admission	Prior COVID infection
1	F	16 years	PIMS-TS	Previously fit and well	COVID-19 PCR **−** SARS-CoV-2 IgG **+**	COVID swab negative at local hospital and on admission
2	M	11 years	PIMS-TS	Previously fit and well	COVID-19 PCR **−** SARS-CoV-2 IgG **+**	SARS-COV-2 PCR + 1 month before admission
3	F	15 years	PIMS-TS	Previously fit and well	COVID-19 PCR **−** SARS-CoV-2 IgG +	Not reported
4	M	10 years	PIMS-TS	Previously fit and well	COVID-19 PCR **–** SARS-CoV-2 IgG **+**	COVID-19 + 1 month prior to admission
5	M	11 years	PIMS-TS	Previously fit and well	COVID-19 PCR **+** SARS-CoV-2 IgG **+**	COVID-19 + 2 months prior to admission
6	F	11 years	COVID-19	COVID pneumonitis, cerebral palsy, epilepsy, quadriplegic	COVID-19 PCR **+** SARS-CoV-2 IgG **+**	COVID-19 sick contact in family
7	F	13 years	COVID-19	Myocarditis secondary to COVID-19, primary COVID-19 infection	COVID-19 PCR **+** SARS-CoV-2 IgG **−**	COVID-19 sick contact in family
8	F	15 years	COVID-19	Pneumonitis, Down syndrome, hypothyroidism, long term ventilation	COVID-19 PCR + SARS-CoV-2 IgG **−**	Not reported
9	F	13 years	COVID-19	Previously fit and well	COVID-19 PCR **+** SARS-CoV-2 IgG not tested	Not reported
10	M	10 years	COVID-19	Dilated cardiomyopathy, Asthma	COVID-19 PCR **+** SARS-CoV-2 IgG **+**	Not reported
11	F	6 months	COVID-19	COVID Pneumonia, Oesophageal atresia, congenital hypothyroidism	COVID-19 PCR **+** SARS-CoV-2 IgG not tested	SARS-COV-2 PCR + 1 month before admission
12	F	10 days	COVID-19	COVID Pneumonia. Previously fit and well	COVID-19 PCR **+** SARS-CoV-2 IgG not tested	COVID-19 sick contact in family
13	F	2 years	COVID-19	Community-acquired Pneumonia. Previously fit and well	COVID-19 PCR **+** SARS-CoV-2 IgG **−**	COVID-19 sick contact in family

IgG: Immunoglobulin G; **+**: positive test; **−**:negative test; F: female; M: male.

### Flow cytometry

PBMC were stained as described [36] using combinations of directly conjugated antibodies supplied by BioLegend or eBioscience listed in Supplementary Table 1 and analysed on an Accuri C6 flow cytometer (BD Biosciences). Data were analysed using FlowJo 10.4.2 (Tree Star). Representative staining is shown in Supplementary Fig. 1. Statistical analyses were carried out using GraphPad Prism software.

### Quantitative RT-PCR

RNA extraction, cDNA synthesis and Q-RT-PCR were as described [37] using *HPRT* for template quantification and normalization. RNA was extracted by PicoPure RNA isolation kit (Applied Biosystems) according to the manufacturer’s instructions. RNA concentration was assessed using a Nanodrop. *ACE2* and *HPRT* were detected using Quantitect primers (Qiagen).

### Isolation of TEC

For isolation of TEC, thymus tissue was cut into small pieces (- 5 mm × 5 mm) and agitated in 50 ml falcon tubes by rolling for 20 minutes in RPMI 10% FBS to remove some thymocytes. It was then incubated for 15 minutes at 37°C with 2–5 ml of digestion buffer containing RPMI + 10% FBS, 250 mg/ml Liberase and 0.5 mg/ml DNase1 (Roche Diagnostics UK). Every 10 minutes thymus pieces were gently passed through a 1 ml pipette. An additional 0.5 mg/ml of DNase1 was added and the tissue was incubated for 10 minutes, after which 0.5 M EDTA was added to the tube, which was topped up with medium to 15 ml and centrifuged. The cell suspension was used for the enrichment of TEC using the EasySep^TM^ Human EpCAM Positive Selection Kit II (StemCell Technologies) to isolate EpCAM + cells.

### Preparation of thymus explants

The thymus was prepared and cultured, as described [38]. Briefly, thymus tissue cut into ~1 mm cubes, was placed on 0.8 μm Millipore filters (Millipore, Massachusetts, US) floating on 400 µl AIMV (Invitrogen, USA) before transfer to Category three tissue culture facilities.

### Virus propagation and plaque assay

The SARS-CoV-2 isolate hCoV-19/England/2/2020 was obtained from Public Health England, and propagated in the Vero E6 cell line, as described previously [39]. A mock (control) condition was conducted in parallel in which an equivalent volume of PBS was used instead of viral inoculum. The viral and mock-inoculated cell media were collected after 48 hours, centrifuged at 10 000 ×*g* for 10 minutes to remove cellular debris and stored at −70°C. Viral titre was determined by plaque assay, as described previously [39]. To calculate the infectious viral load, the number of plaques was multiplied by the dilution factor, yielding the final plaque count per millilitre (pfu/ml).

### Viral infection of human thymus explants

Thymus explants were transferred to 96 well plates: after rinsing with sterile Dulbeccos’ Phosphate buffered saline with calcium chloride and magnesium chloride (PBS++) they were positioned on 0.8 μm Millipore filters above 150 µl AIMV. Wells were inoculated by dropwise addition directly on top of the tissue of 50 µl SARS-CoV-2 viral inoculum suspended in PBS++ (1 × 10^5^ pfu ml^−1^, ~0.1 MOI) or an equivalent volume of mock inoculum suspended in PBS++ (mock infection) and cultured at 37°C, 5% CO_2_ for up to 48 hours. At 24 and 48 hours, samples were withdrawn from the media for analysis in plaque assays. Media-only controls were also inoculated with SARS-CoV-2 inoculum and analysed by plaque assay at 24 and 48 hours.

### SARS-CoV-2 sgRNA RT-PCR

Subgenomic RNA (sgRNA) is only transcribed in infected cells and indicates the presence of active replication [40]. RNA was extracted from human thymus cultures using the Arcturus PicoPure RNA isolation kit (Applied Biosystems) and converted to cDNA using the High-capacity cDNA reverse transcription kit (Applied Biosystems), following manufacturers’ instructions. cDNA was tested for the presence of Envelope (E) and Nucleocapsid2 (N2) sgRNA using published primers (Thermofisher) and Q-RT-PCR protocols [41, 42], with modifications described in Supplementary Table 2. The cycle threshold cut-off for negative samples (undetected virus) was considered 38 cycles.

### TREC analysis

DNA was extracted from 2 × 10^4^ PBMC from each individual for TREC analysis. Cells were centrifuged at 3600 rpm for 20 minutes at 4°C. Then, 20 μL of lysis Buffer (KCL 50 mM, Tris-HCL (pH 8.5) 10 mM, MgCl_2_ 1.5 mM, Gelatine 0.01% v/v, Tween20 0.45% v/v and NP40 0.45% v/v) and 4 μl of 5 mg/ml of Proteinase K were added to the pellet and incubated for 1 hour at 56°C, then 15 minutes at 95°C and DNA was then stored at −20°C before TREC analysis. Signal joint (Sj) TREC analysis was assessed by quantitative PCR, as described [43]. Briefly, test DNA from PBMC was amplified using primers and probes specific for TREC and *TRA* constant region (*TRAC*) alongside a standard curve. TREC were normalized against *TRAC* levels to present data as TREC/10^6^ PBMC (assuming two copies of *TRAC* per diploid cell).

### Cell sorting for TCR repertoire analysis

Frozen PBMC from children (age 10–16 years) with COVID-19, PIMS-TS, and paediatric healthy controls were thawed and stained for DAPI, CD3PE, CD4PercpCy5.5 and CD8APC and sorted at the GOSICH Flow Cytometry Facility using FACS AriaIII (BD). Cells stained as DAPI-CD3+CD4+ or DAPI-CD3+CD8+ were collected in AIM-V, pelleted and resuspended in an extraction buffer from Arcturus PicoPure RNA isolation kit (Applied Biosystems) and kept at −80°C for RNA extraction.

### TCR amplification and sequencing

TCR amplification and sequencing were as described [18, 44, 45]. Samples were sequenced on the NextSeq using the NextSeq 500/550 Mid Output Kit v2.5 (300 Cycles; Illumina, cat. no: 20024905) at UCL Genomics.

### Error correction and outputting

The NextSeq outputs files in the format named binary-based call (.bcl) were converted into FASTQ files using bcl2fastq for downstream processing in Python 2.7 using a pipeline of scripts described previously [44, 46]: *Decombinator_v3.1* (available at: https://github.com/innate2adaptive/Decombinator/).

### Downstream analysis of repertoire sequencing data

For further analyses, *R Studio* was used. The *tidyverse* set of packages were used for data manipulation and visualizations, in particular *ggplot2* [47, 48]. Dotplots show mean ± c.i (package *ggpubr* [49]) and statistical comparisons were carried out by unpaired Student’s *t*-test or Welch’s *t*-test as appropriate using the package *rstatix* [50]. *Pheatmap* and *ComplexHeatmap* were used to generate Pearson correlation heatmaps [51, 52].

#### TCR abundance distribution

TCR abundance (number of copies of each sequence) was plotted against their proportion of the repertoire in a log–log plot. This transformed distribution follows approximately a linear distribution apart from the largest clonotypes and can be fitted to a discrete power law ($f\left(x\right)=kx^{-\alpha }$) as seen in previous studies [44, 53]. The TCR abundance frequency was fitted to a discrete power law using maximum likelihood estimation [54, 55] using the *PoweRLaw* package [56]. Power law exponents (α) were then plotted and compared.

#### Diversity indices

Before calculating diversity indices, α and β identified TCRs or CDR3s were subsampled (rarefied) to a number lower than the smallest repertoire using the package *vegan* [57] to correct for differences in diversity due to sample size. The mean diversity indices (Shannon Entropy, Gini Index) were then calculated from 1000 repeats of this random sampling. The Shannon Entropy was computed using the package *vegan* [57]. Gini index was computed using *ineq* package [58].

#### Principle Component Analysis

Raw counts were log_10_ transformed to account for differences in sample size, using a pseudocount of 0.01 for any counts that were not observed. Z-scores were then calculated from these values by subtracting the mean and dividing by the standard deviation. Principal component analysis (PCA) was then computed using the base R function *prcomp* and *factoextra* package [59].

## Results

### Thymus tissue can be infected with SARS-CoV-2 *in vitro*

**Figure 1:**
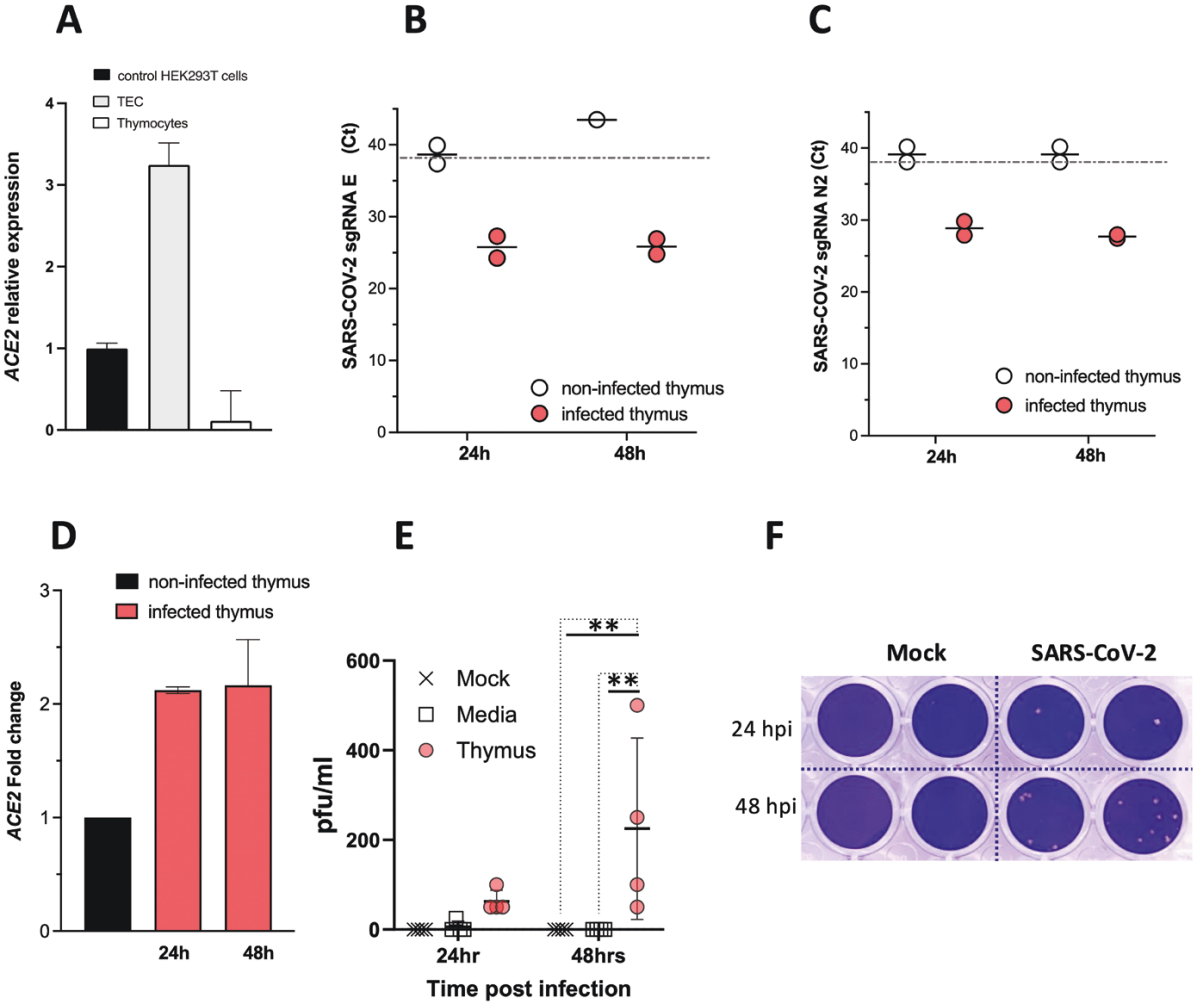
Human thymus tissue can be infected by SARS-CoV-2 *in vitro*. **(A**) Relative gene expression of *ACE2* in paediatric thymus tissue (Q-RT-PCR). Values shown are mean±SEM relative expression in enriched thymus epithelial cells (TEC) and thymocytes compared to control HEK293T cells. (**B**-**E**) Human thymus explants were cultured with SARS-CoV-2 or without (mock) for up to 48 hours. (**B-C**) Q-RT-PCR to detect subgenomic (sg) RNA from SARS-CoV-2 from thymus tissue, for Envelope (E) (**B**) and Nucleocapsid 2 (N2) (**C**), giving cycle threshold (Ct) values for samples in which sgRNA was detectable in infected thymus (orange dots) or not detectable in mock thymus (white dots) at 24 and 48 hours. The line shows the cut-off Ct value for detection at 38 cycles. (**D**) Fold change gene expression of *ACE2* by Q-RT-PCR, showing mean±SEM in thymus cultured with SARS-CoV-2 (orange) or without (black). (**E**) Infectious viral load in surrounding media (pfu/ml) from SARS-CoV-2 infected thymus (MOI 0.1) (orange dots), with media, only inoculated with SARS-CoV-2 (black crosses), and mock-infected thymus controls (open squares) at 24 and 48 hours. Two-way ANOVA with Tukey’s multiple comparisons test. Individual values with mean and SD are given (*n* = 4); **P < 0.01. (**F**) Representative plaque assay at 24 and 48 hours post-infection (hpi).

We first investigated if thymus tissue could be infected by SARS-CoV-2. We examined the expression of SARS-CoV-2 receptor molecule *ACE2* by Q-RT-PCR in the paediatric human thymus, comparing expression levels in purified TEC and thymocytes to levels in control human embryonic kidney (HEK2983T) cells. *ACE2* was expressed in TEC but not detected in thymocytes (Fig. 1A). We then tested if thymus tissue could be infected by SARS-CoV-2 *in vitro*. Thymus explants were cultured for up to 48 hours in the presence of SARS-CoV-2 or “mock” control inoculum. To determine if the thymus tissue was infected, we carried out Q-RT-PCR for the presence of subgenomic (sg) SARS-CoV-2 Envelope (E) and Nucleocapsid 2 (N2) RNA, as these sgRNA species are only transcribed when a mammalian cell is infected with the virus, but are not present in the viral particles [40]. At 24 and 48 hours after inoculation, sgRNA for E and N2 were detected in the thymus explants inoculated with SARS-CoV-2, but not in control thymus cultures (with “mock” inoculum; Fig. 1B-C). In support of this, *ACE2* expression was increased by more than two-fold in thymus explants exposed to SARS-CoV-2 but not in mock-infected controls (Fig. 1D). To test if thymus tissue can propagate SARS-CoV-2 we carried out plaque assays with medium removed from the SARS-CoV-2-inoculated thymus cultures at 24 and 48 hours, in comparison to wells inoculated with SARS-CoV-2 in absence of tissue, and wells containing thymus explants with “mock” inoculum. After 48 hours, live SARS-CoV-2 was detected in the SARS-CoV-2-inoculated thymus cultures, but not in either mock-inoculated thymus cultures or the SARS-CoV2-inoculated medium-only cultures (Fig. 1E-F). Viral titres (Pfu/ml) detected in the thymus cultures; however, were lower than those measured in SARS-Co-V2-infected nasal epithelial cell cultures [39]. These data confirm findings from a recent study showing that mTEC expresses ACE2 and that purified TEC can be infected by SARS-CoV2 *in vitro* when cultured on irradiated feeder cells [35]. We additionally showed that whole thymus tissue (which contains many cell types including TEC, thymocytes, macrophages and fibroblasts) can be infected and produce SARS-CoV2 particles at 48 hours after infection, although we did not identify which cell type within the explants was propagating viral particles. This experiment thus indicates that SARS-CoV-2 infection of the thymus could impact T-cell development.

### T-cell populations in the blood of children with PIMS-TS and COVID-19

**Figure 2:**
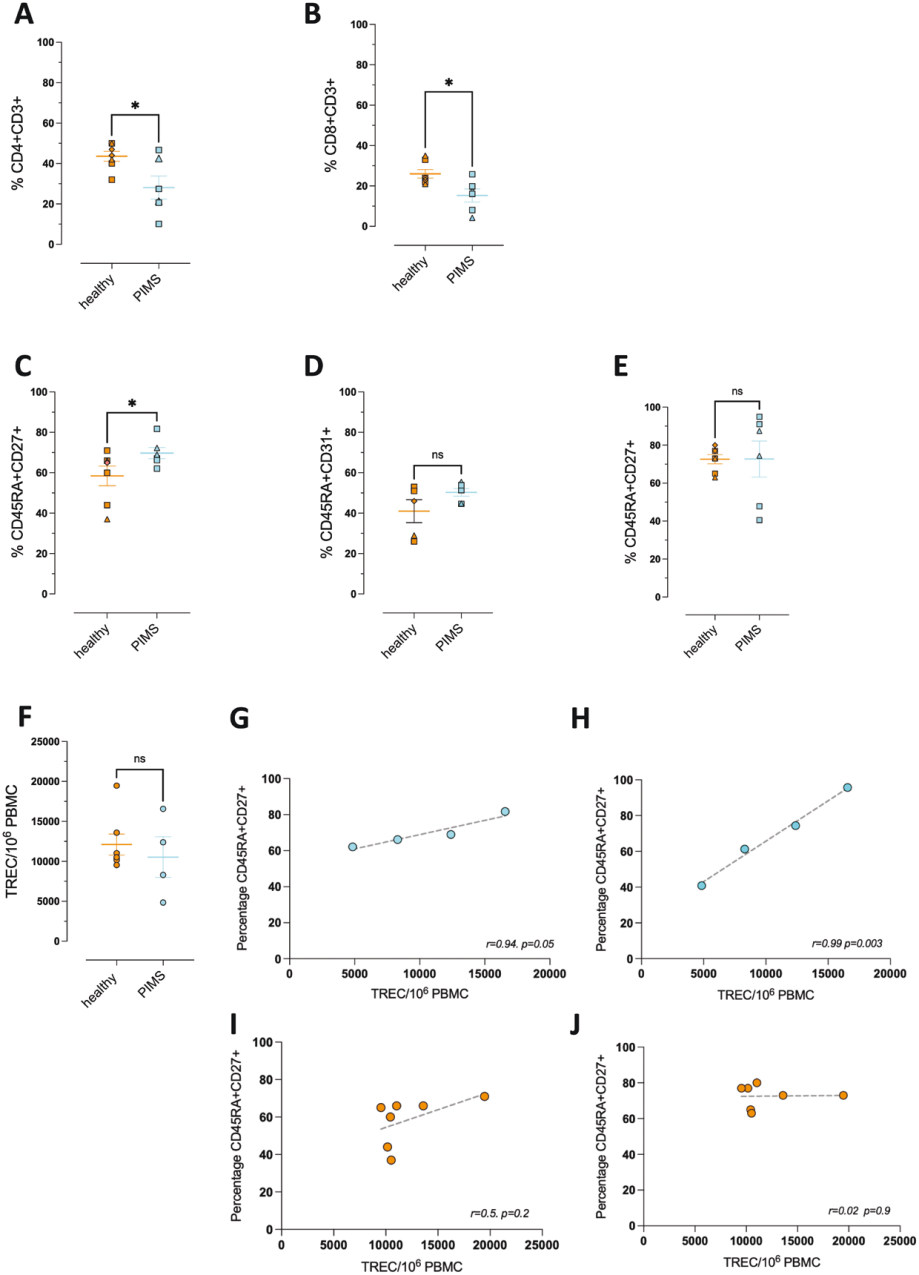
Lymphocyte populations in the blood of paediatric age-matched healthy control, and PIMS-TS patients. **(A-E)** Percentages of T cells, naïve T cells and recent thymic emigrants (RTE) in PBMC prepared from blood from paediatric healthy control (healthy, orange) and PIMS-TS (PIMS, light blue) patients in the age range 10–16 years old, determined by flow cytometry, as shown in SFig1. (**A**) CD4+ CD3+ (**B**) CD8+CD3+. (**C-E**) percentages are gated on CD4+CD3+ (**C-D**) and CD8+CD3+ (**E**). (**C**): CD45RA+CD27+ (naïve CD4); (**D**): CD45RA+CD31+ (CD4 RTE); (**E**) CD45RA+CD27+ (naïve CD8). Each dot represents an individual child with females represented by triangles and males as squares. Plots show mean ± SEM.; *P < 0.05. Mann–Whitney *U* test. **(F)** The plot shows the number of signal joint TREC per 10^6^ PBMC in blood samples from healthy control and PIMS-TS groups, determined by quantitative PCR. (**G-H**) Plots show the number of TREC/10^6^ PBMC (x-axis) plotted against the proportion of naïve (CD45RA+CD27+) cells (y-axis), gated on CD4+CD3+ (**G** and **I**) and on CD8+ CD3+ (**H** and **J**), for PIMS-TS (**G-H**) and healthy controls (**I-J**). Each point represents an individual child. Lines show best fit. Pearson’s correlation coefficient (*r*) and *P* value are given.

As we were unable to obtain thymus biopsies from PIMS-TS patients, we compared T-cell populations in PBMC from blood samples obtained from hospitalized paediatric patients, diagnosed with PIMS-TS, compared to healthy children in the corresponding age range (10–16 years; median age 13 years; Fig. 2A-F, Supplementary Fig. 1). The proportions of CD4+CD3+ and CD8+CD3+ T cells in PBMC were reduced in the PIMS-TS patients compared to age-matched healthy children (Fig. 2A-B). We further characterized T-cell populations by expression of CD45RA, CD27, and CD31, to identify naïve T cells and CD4 recent thymic emigrants (RTE) [43, 60, 61]. Gating on the CD4+CD3+ population, the percentage of naïve cells (CD45RA+CD27+), was increased in the PIMS-TS patients compared to healthy children (Fig. 2C), but we found no differences in mean proportions of CD4 RTE (CD45RA+CD31+) or naïve (CD45RA+CD27+) cells within the CD8 population (Fig. 2D-E).

Signal joint TCR excision circles (TREC) are produced during TCR gene rearrangement in the thymus but diluted by cell division, so provide a reliable measure of recent thymic output. TREC levels in PBMC were similar between healthy and PIMS-TS groups, despite the fact that the PIMS-TS samples contained a lower proportion of T cells than healthy controls (Fig. 2A-B, F). In PIMS-TS samples, the number of TREC/10^6^ PBMC correlated positively and closely to the proportion of CD4 cells that were naïve (CD45RA+CD27+; *r* = 0.94, *P* = 0.05); with additionally almost perfect positive correlation to the proportion of CD8 cells that were CD45RA+CD27+ (*r* = 0.99, *P* = 0.003; Fig. 2G-H). This correlation was not observed in samples from healthy children, where TREC numbers did not correlate strongly or significantly with the proportion of naïve CD4 cells (*r* = 0.5, *P* = 0.2) or naïve CD8 cells (*r* = 0.02, *P* = 0.9; Fig. 2I-J). This difference indicates that while in the healthy children of this age range, the naïve T-cell pool is made up of both RTE and cells that have arisen as a result of peripheral homeostatic expansion, in the PIMS-TS group, the naïve T-cell population is made up of cells that contain TREC (RTE).

### TCR repertoire in blood T cells from healthy, PIMS-TS and COVID-19 groups

We next investigated the TCR repertoire in blood T cells from PIMS-TS patients, healthy children, and also children who were admitted to GOSH PICU diagnosed with severe COVID-19, in the 10–16 age range, as an additional nonhealthy control for children who were admitted to the same PICU, infected with SARS-CoV-2. Blood samples from the COVID-19 patient group in this age range contained lower proportions of CD4+ CD3+ and CD8+CD3+ in PBMC than healthy samples, but we found no significant differences in the proportions of naïve or CD4+ RTE cells (Supplementary Fig. 2A-E).

We sequenced the TCRβ and TCRɑ chain repertoires separately from rearranged transcripts: following RNA extraction from FACS-sorted CD4+CD3+ and CD8+CD3+ cells we used a TCR sequencing protocol and analysis pipeline which includes single-strand DNA ligation that tags each molecule of TCRβ and TCRɑ chain mRNA with a unique molecular identifier, allowing for PCR bias correction in analysis [44, 45, 62]. To visualize the frequency distribution of the TCRβ and TCRɑ repertoires in each population, we plotted the TCR abundance (number of copies of each sequence) against their proportion of the repertoire, and fitted these distributions of TCRβ and TCRɑ abundances to a discrete power law, where the power law exponent corresponds to the gradient on the log–log plots ( Supplementary Fig. 3A). We found no differences in power law exponent for either TCRβ or TCRɑ repertoires between the three groups of children for blood CD4 and CD8 T-cell populations (Supplementary Fig. 3B-C), indicating that the distribution of the TCR repertoire within the T-cell populations was broadly unaltered in COVID-19 and PIMS-TS. To confirm this, we calculated the proportion of the TCRβ and TCRɑ repertoires represented by the top 5% most abundant TCRβ or TCRɑ clonotypes in each population and found no difference between groups in either CD4 or CD8 T cells (Supplementary Fig. 3D-E). When we calculated the number of TCRɑ and TCRβ sequences detected above the frequency threshold (top 5% most abundant sequences) we again observed no significant differences in the mean abundance of individual sequences between the three groups (Supplementary Fig. 3F-G). Comparison of standard indices of diversity and richness (Shannon entropy [63]) and distribution (Gini index [64]) also showed no differences between the three groups (Supplementary Fig. 3H-K), confirming that while the PIMS-TS and COVID-19 groups both had a lower overall proportion of CD4 and CD8 T cells in blood PBMC, their TCRɑ and TCRβ repertoires had equivalent diversity and distribution to that of healthy children.

### Differences in TCR V and J gene segment usage and CDR3 non-template additions between healthy, PIM-TS and COVID-19 groups

**Figure 3:**
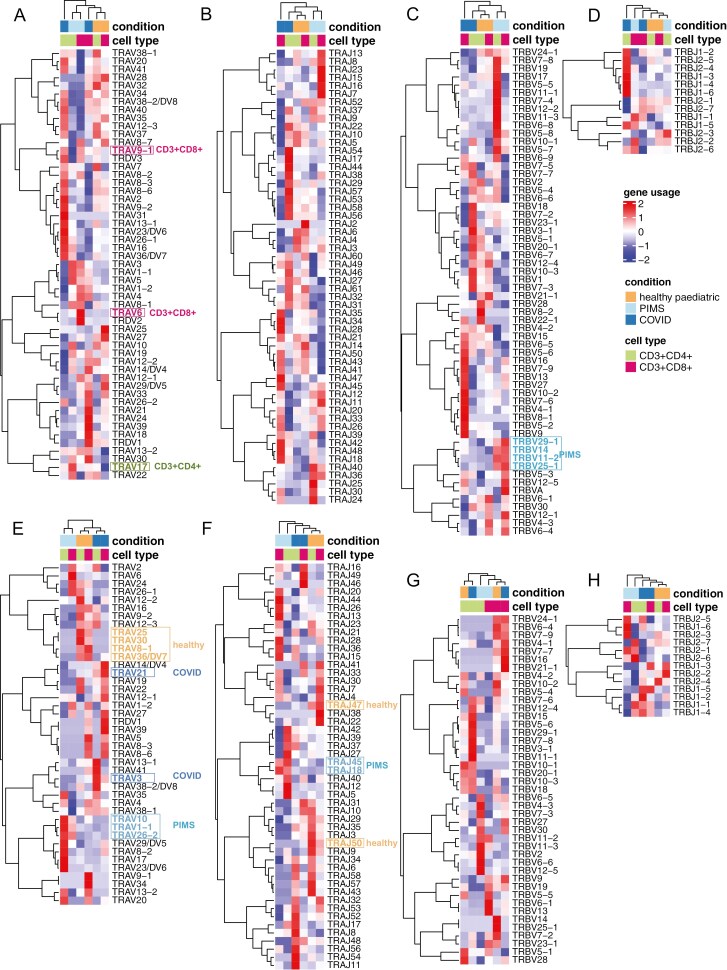
PIMS-TS and COVID-19 patients’ repertoires favour distinct V and J gene segments. TCR ɑ-chain (**A-B**, **E-F**) and β-chain (**C-D**, **G-H**) repertoires were sequenced from FACS-sorted CD3+CD4+ and CD3+CD8+ populations from PBMC isolates from 10–16 year age range healthy controls (healthy paediatric, orange, *n* = 4/5), PIMS-TS patients (PIMS, light blue, *n* = 4/5) and COVID-19 patients (COVID, dark blue, *n* = 5). CD3+CD4+ and CD3+CD8+ populations are coloured green and pink respectively. (**A-H**) Heatmaps of proportional α-chain variable (V) (**A, E**), α-chain joining (J) (**B, F**), β-chain V(**C, G**) and β-chain J (**D, H**) gene usage of all TCRs (**A-D**) and for the top 0.1% most abundant sequences (**E-H**) for healthy controls, PIMS-TS and COVID-19 patients in CD3+CD4+ and CD3+CD8+ populations. Each column represents a mean of 4 or 5 patients and was clustered using Euclidian distance, while each row represents a gene segment and was clustered using Pearson correlation.

We compared V and J gene segment usage in CD4 and CD8 populations between the three groups of children (Supplementary Fig. 4-5) and visualized this as heat maps of mean proportional gene segment usage (Fig. 3A-D). The V region of the β-chain (TRBV), and the J region of the ɑ-chain (TRAJ) samples clustered first by condition, before cell type, with the healthy group clustering more closely to the COVID-19 group than to the PIMS-TS group (Fig. 3B-C). In the PIMS-TS group, several TRBV gene segments, such as TRBV29-1, TRBV14, TRBV11-2, and TRBV25-1, were overrepresented in both CD4 and CD8 populations (Fig. 3C). For the α-chain V region gene segments, the healthy samples for CD4 and CD8 T cells clustered together, as did the PIMS-TS samples, but not COVID-19 samples (Fig. 3A). The analysis also revealed some gene segment usage that appeared overrepresented in different populations, such as TRAV6 and TRAV9-1 in the PIMS-TS CD8 T-cell populations, and TRAV17 in the PIMS-TS CD4 T-cell populations.

Comparison of TCR gene usage in the top 0.1% most abundant sequences showed that both TCRɑ gene segments (TRAV and TRAJ) and TRBJ now clustered by condition, with several gene segments overrepresented in healthy repertoires (TRAV25, TRAV30, TRAV8-1, TRAV36/DV7, TRAJ47, TRAJ50), COVID-19 repertoires (TRAV21, TRAV3), and PIMS-TS repertoires (TRAV10, TRAV1-1, TRAV26-2, TRAJ45 and TRAJ18; Fig. 3E, F and H). In contrast, TRBV clustered first by cell type in the top 0.1% most abundant sequences (Fig. 3G). Thus, disease state had a greater influence on TRBV in unexpanded clones, whereas its influence on TRAV, TRAJ and TRBJ was greater in expanded clones.

### PIMS-TS and Healthy TCRβ and TCRɑ repertoires use distinct combinations of V and J segments

**Figure 4:**
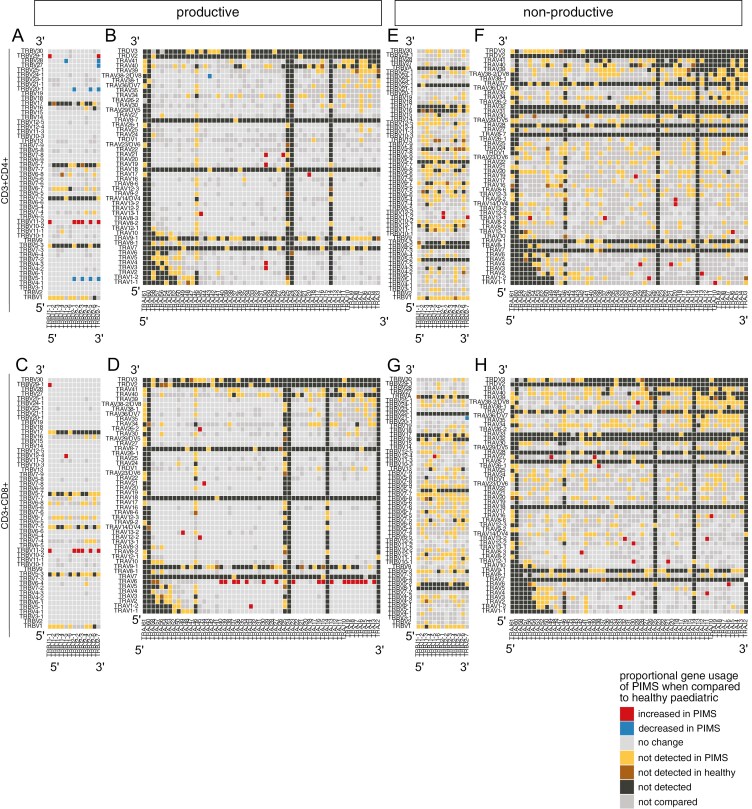
Proportional combinatorial VxJ gene usage differs between healthy controls and PIMS-TS patients TCRɑ- and β-chain repertoires sequenced from FACS-sorted CD3+CD4+ and CD3+CD8+ populations from PBMC isolated from healthy paediatric controls (healthy paediatric, *n* = 4/5), and PIMS-TS patients (PIMS, *n* = 4/5) in 10–16 year age range. (**A-D**) Plots show β-chain (left column, **A** and **C**) and α-chain (right column, **B** and **D**) proportional VxJ gene usage of productive TCR sequences for PIMS-TS patients compared to healthy controls in CD3+CD4+ (**A** and **B**) and CD3+CD8+ (**C** and **D**) populations. (**E-H**) Plots show β-chain (**E** and **G**) and α-chain (**F** and **H**) proportional VxJ gene usage of non-productive TCR sequences for PIMS-TS patients compared to healthy controls in CD3+CD4+ (**E** and **F**) and CD3+CD8+ (**G** and **H**) populations. Gene segments are listed in the order they appear on the chromosome from top to bottom for V region genes and left to right for J region gene segments, as indicated. Red tiles signify increased usage of V × J combinations in PIMS-TS patients compared to healthy controls (*P *< 0.05), while blue tiles signify decreased usage in PIMS-TS patients (*P *< 0.05) compared to healthy controls. Light grey tiles signify no change in gene usage (*P *> 0.05), yellow tiles signify V × J combinations not detected in PIMS-TS patients; brown tiles signify V × J combinations not detected in healthy controls; and black tiles signify V × J combinations not detected in either PIMS-TS patients or healthy controls. Only combinations that were detected in at least three patients per group were compared. Dark grey tiles signify combinations that were not compared. Statistical comparisons were carried out by unpaired Student’s *t*-test followed by FDR-adjustment of *P* values (5%, Benjamini–Hochberg procedure) for multiple testing.

The CDR3 region of the TCR determines TCR antigen-specificity and is encoded by the junction between V and J gene segments and non-template insertions and deletions so that specificity is determined by both combinatorial V × J usage and junctional diversity. As PIMS-TS repertoires showed distinctive patterns of V and J gene usage, we compared the combinatorial proportional V × J use in productive TCRβ and TCRɑ repertoires in CD4 and CD8 T cells from PIMS-TS and healthy groups, and visualized this by plotting V × J combinations according to the chromosomal position of the gene segments (Fig. 4A-D). For the CD4 TCRβ repertoires, 8 V × J combinations were significantly higher in the PIMS-TS group, using just two Vβ segments (TRBV29-1 and TRBV11-2) in combination with 6 TRBJ segments (Fig. 4A). For the CD8 TCRβ repertoires, 7 V × J combinations were significantly higher in the PIMS-TS group, using just three Vβ segments (TRBV29-1 and TRBV11-2, as seen in the CD4 repertoires, and additionally TRBV12-4) in combination with 6 TRBJ segments, with no correlation to chromosomal location or TRBJ-cluster (Fig. 4C).

For proportional productive TCRɑ V × J combinations, in CD4 cells, 7 combinations were increased in the PIMS-TS group, using 6 different V region genes and 4 different J gene segments (Fig. 4B). For CD8 cells, 21 combinations were increased in the PIMS-TS group, using 20 different TRAJ segments, but only 6 TRAV segments (16 combinations used TRAV6; Fig. 4D). The V × J combinations that showed increased proportional usage showed enrichment of 5′ TRAV segments.

### Combinatorial proportional VJ use in non-productive TCRβ and TCRɑ repertoires from Healthy and PIMS-TS groups

The analysis of combinatorial proportional productive VJ usage revealed biases in PIMS-TS V × J combinations for both TCRβ and TCRɑ. Enrichment of given V × J combinations could result from the peripheral disease process, in which TCRs with particular specificities are expanded during infection or inflammation, or from TCR selection in the thymus, that might favour particular V × J combinations for MHC-restriction during repertoire selection. Alternatively, these enrichments of V × J combinations could be the result of differences in gene rearrangement during T-cell development in the thymus.

To distinguish between differences in gene rearrangement and differences in the TCR protein’s binding specificity we therefore compared combinatorial proportional V × J usage in non-productive TCRβ and TCRɑ repertoires in CD4 and CD8 T cells from PIMS-TS and healthy groups (Fig. 4E-H). Although most out-of-frame (non-productive) rearrangements generated during TCR gene rearrangement are eliminated by nonsense-mediated decay during T-cell development [65], some non-productive TCR transcripts persist and analysis of these sequences has been used to investigate the V(D)J recombination process [66–68].

In non-productive CD4 TCRβ repertoires, we identified 3 V × J combinations that were increased in the PIMS-TS group compared to the healthy group. Interestingly, two of these rearrangements (TRBV11-2 × TRBJ2-1 and TRBV11-2 × TRBJ2-7) were also enriched in the productive PIMS-TS group (Fig. 4A and E). Likewise, in the CD8 TCRβ comparison, the only non-productive V × J rearrangement that was higher in the PIMS-TS group (TRBV12-4 × TRBJ1-5) was also higher in the productive PIMS-TS repertoires (Fig. 4C and G).

Comparison of the non-productive proportional TCRɑ V × J combinations between PIMS-TS and healthy groups showed that >400 combinations that were not detected in the CD4 PIMS-TS group were present in healthy controls, whereas 11 V × J combinations were higher in the PIMS-TS group (Fig. 4F). V × J combinations that were increased in PIMS-TS samples in general used distal rearrangements (5′TRAV × 3′TRAJ). The non-productive CD8 TCRɑ combinations showed a similar pattern. While >400 V × J combinations were not detected in the PIMS-TS group that were present in the healthy group, their frequency was reduced in the distal quadrant of the plot (32 in the 5′V to 3′J quadrant compared to 110 in the 3′V to 5′J quadrant). Likewise, the 15 V × J combinations that were increased in the PIMS-TS group were enriched for distal 5′ TRAV to 3′ TRAJ rearrangements (Fig. 4H).

Thus, analysis of the rearrangements of non-productive sequences showed differences in gene rearrangements that must have taken place in the thymus. The *TRA* locus can undergo multiple rounds of rearrangement on each chromosome, moving 3′ to 5′ along the series of TRAV gene segments, while moving 5′ to 3′ along the series of TRAJ segments. Proximal pairs (3′TRAV segments with 5′TRAJ segments) initiate this sequence of rearrangements [15] and rearrangements of distal pairs (5′TRAV with 3′TRAJ segments) take place later in time after initiation of *TRA* locus rearrangement in a given cell. Circumstances in which thymocytes are produced rapidly, such as after depletion of the thymus following hydrocortisone treatment, produce more proximal 3′TRAV to 5′TRAJ rearrangements [18]. This suggests that the bias towards distal 5′TRAV to 3′TRAJ rearrangements observed in the non-productive PIMS-TS sequences may reflect slower differentiation of individual thymocytes, allowing more time for these distal rearrangements to take place.

### Comparison of combinatorial proportional V × J use in productive and non-productive TCRβ and TCRɑ repertoires between COVID-19 and healthy groups

**Figure 5:**
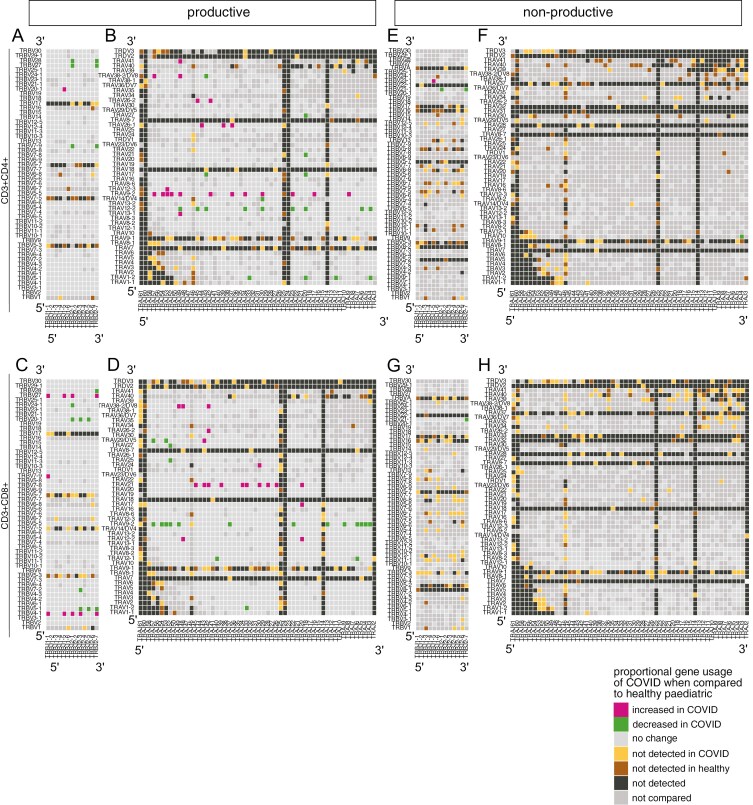
Proportional combinatorial V × J gene usage differs between healthy controls and COVID-19 patients. TCRɑ and TCRβ repertoires sequenced from FACS-sorted CD3+CD4+ and CD3+CD8+ populations from PBMC isolated from healthy paediatric controls (healthy paediatric, *n* = 4/5), and COVID-19 patients (COVID, *n* = 5) in 10–16 year age range. (**A-C**) Plots show β-chain (left column, **A** and **C**) and α-chain (right column, **B** and **D**) proportional V × J gene usage of productive TCRs for COVID-19 patients compared to healthy controls in CD3+CD4+ (**A-B**) and CD3+CD8+ (**C-D**) populations. (**E-H**) Plots show β-chain (**E** and **G**) and α-chain (**F** and **H**) proportional V × J gene usage of non-productive TCRs for COVID-19 patients compared to healthy controls in CD3+CD4+ (**E** and **F**) and CD3+CD8+ (**G and H**) populations. Gene segments are listed in the order of their location on the chromosome from top to bottom for V region genes and left to right for J region gene segments, as indicated. Pink tiles signify increased usage of V × J combinations in COVID-19 patients compared to healthy controls (*P *< 0.05), while green tiles signify decreased usage in COVID-19 patients (*P *< 0.05) compared to healthy controls. Light grey tiles signify no change in gene usage (*P *> 0.05), yellow tiles signify V × J combinations not detected in COVID-19 patients; brown tiles signify V × J combinations not detected in healthy controls; and black tiles signify V × J combinations not detected in either COVID-19 patients or healthy controls. Only combinations that were detected in at least three patients per group were compared. Dark grey tiles signify combinations that were not compared. Statistical comparisons were carried out by unpaired Student’s *t*-test followed by FDR-adjustment of *P* values (5%, Benjamini–Hochberg procedure) for multiple testing.

The COVID-19 group provides additional non-healthy control to assess the impact of SARS-CoV-2 on thymus function, so we also compared combinatorial proportional V × J use in productive and non-productive TCRβ and TCRɑ repertoires between COVID-19 and healthy groups (Fig. 5A-H). The productive COVID-19 CD4 repertoires showed proportionally increased usage of only one combination (TRBV20-1 × TRBVJ1-5), which was not identified in the non-productive analysis (Fig. 5A and E). In the productive COVID-19 CD8 repertoires we found increased proportional usage of 10 V × J combinations, 4 of which used TRBV27, 5 of which used TRBV4-1, and none of which were significantly different in the non-productive sequences, indicating that the disease had selected for expansion of T cells expressing these TCRs (Fig. 5C and G).

We observed many changes in productive proportional combinatorial V × J usage in TCRɑ CD4 repertoires (22 combinations up in COVID-19 group, with enrichment of combinations using TRAV9-2, and 22 combinations down) and CD8 repertoires (19 combinations up in COVID-19 group with enrichment of TRAV21, and 22 combinations down; Fig. 5B and D). Interestingly, we detected no significant increases in non-productive proportional combinatorial TCRɑ V × J usage in either CD4 or CD8 populations (Fig. 5F and H). This is in contrast to the PIMS-TS repertoires and supports the contention that the changes in proportional TCRɑ combinatorial V × J usage observed in the COVID-19 samples are the result of expansion of clonotypes using these TCR gene segments rather than a result of a difference in the gene rearrangement process in the thymus.

### Principal component analysis of combinatorial V-J use separates healthy repertoires from PIMS-TS repertoires on PC1, irrespective of cell type

**Figure 6:**
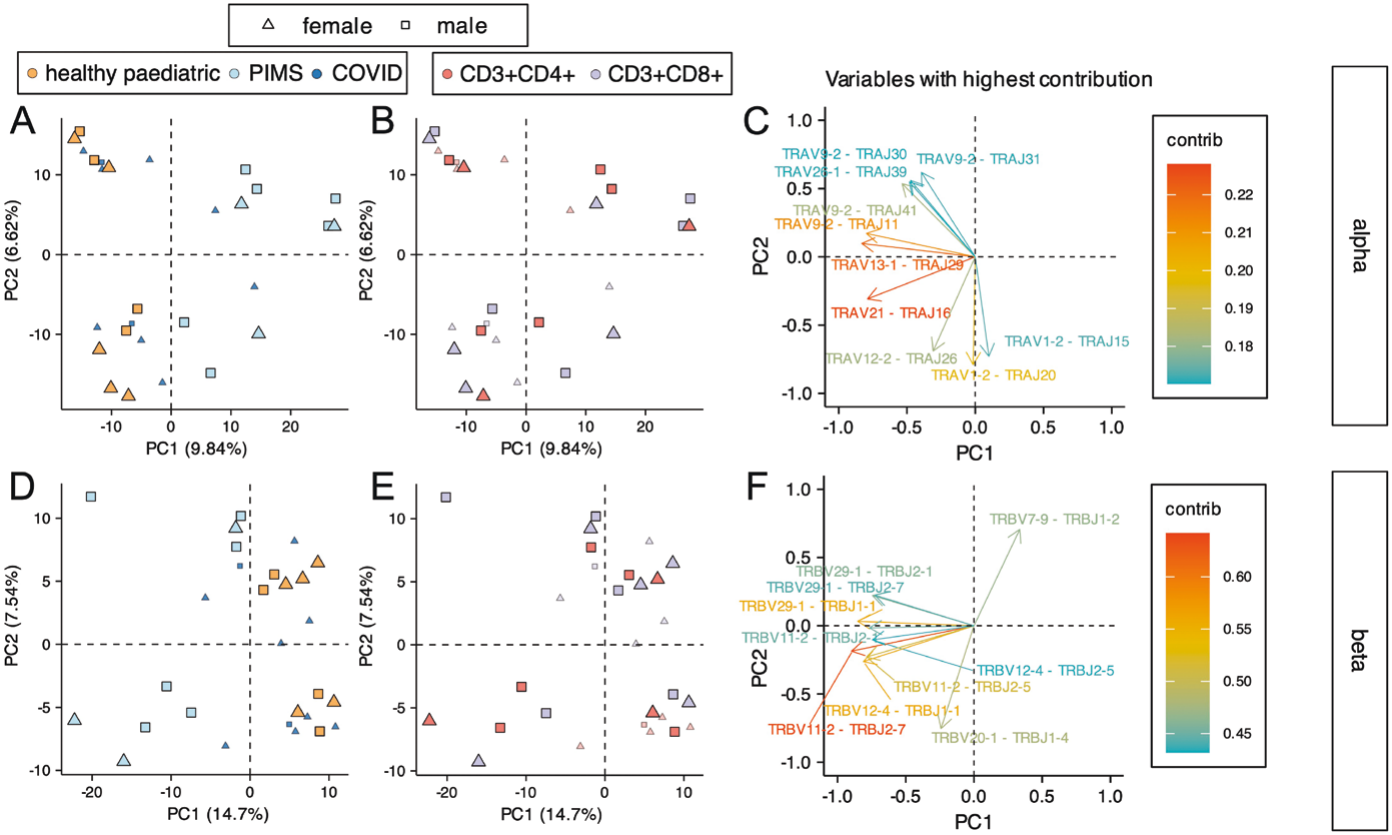
Principal component analysis (PCA) of VxJ combinations in the top 5% most abundant sequences separates PIMS-TS samples from healthy samples for TCRɑ and TCRβ repertoires. TCRɑ- and β-chain repertoires were sequenced from FACS-sorted CD3+CD4+ and CD3+CD8+ populations from PBMC isolated from healthy paediatric controls (healthy paediatric, orange; *n* = 4/5), PIMS-TS patients (PIMS, light blue, *n* = 4/5) and COVID-19 patients (COVID, dark blue; *n* = 5) in 10–16 year age range. Each shape represents an individual repertoire. Squares represent males and triangles represent females. PCA was carried out using counts for each V × J combination from the top 5% most abundant sequences as input. **(A-F)** PCA biplot of ɑ-chain (**A-C**) and β-chain (**D-F**) VxJ frequency distributions from top 5% most abundant sequences for healthy control, PIMS-TS and COVID-19 patients in the CD3+CD4+ and CD3+CD8+ populations, showing PC1 and PC2, where each dot represents an individual repertoire. In (**A** and **D**) healthy control, PIMS-TS and COVID patient repertoires are coloured in orange, light blue and dark blue, respectively, while in (**B** and **E**) CD3+CD4+ and CD3+CD8+ populations are coloured red and grey, respectively. (**C, F**) Top 10 highest V × J combinations that contribute most to PC1 and PC2 of the PCA of ɑ-chain (**C**), and β-chain (**F**) V × J frequency distributions for paediatric control, PIMS and COVID patients in the CD3+CD4+ and CD3+CD8+ populations (shown in **A-E**).

We carried out PCA using the counts of each V × J combination from the top 5% most frequent TCRɑ sequences (Fig. 6A-C) and TCRβ sequences (Fig. 6D-F) for PIMS-TS, COVID-19 and healthy control repertoires. For the TCRɑ chain, PIMS-TS repertoires separated from healthy control repertoires on PC1 irrespective of cell type or sex. All PIMS-TS repertoires fell on the positive side of the axis, and all healthy control and 8 COVID-19 repertoires fell on the negative side of PC1 (Fig. 6A-B). Nine out of the ten combinations that contributed most to the variability fell on the negative axis of PC1, and included TRAV9-2 × TRAJ30 and TRAV26-1 × TRAJ39, which both showed increased proportional usage in the COVID-19 CD4 repertoires compared to healthy controls (Fig. 6B).

For the TCRβ chain, PIMS-TS repertoires separated from healthy controls on PC1 irrespective of cell type and gender. All PIMS-TS and three COVID-19 repertoires fell on the negative side of the axis, and all healthy control and 7 COVID-19 repertoires were positive on PC1 (Fig. 6D-E). Only one of the ten combinations that contributed most to the variability fell on the positive axis of PC1 (TRBV7-9 × TRBJ1-2; Fig. 6F), which also showed increased proportional usage in the COVID-19 CD8 repertoires compared to healthy controls (Fig. 6C). Of the nine out of ten combinations that contributed most to the variability and fell on the negative (PIMS-TS) axis of PC1, six also showed statistically significant increased proportional usage in the PIMS-TS repertoires compared to healthy controls (TRBV11-2 × TRBJ2-7, TRBV11-2 × TRBJ1-1, TRBV11-2 × TRBJ2-5, TRBV11-2 × TRBJ2-1, TRBV29-1 × TRBJ2-7, TRBV29-1 × TRBJ1-1; Fig. 5A and C). Thus, the PCA of the most frequent 5% of sequences clearly showed that the PIMS-TS TCRɑ and TCRβ repertoires used distinct V × J combinations, despite the fact that they displayed normal TCR clonotype distribution (Supplementary Fig. 3D-G).

## Discussion

Our starting hypothesis for this investigation of T cells and the TCR repertoire of paediatric PIMS-TS patients was the idea that while active thymus output in childhood might be protective against severe COVID-19 disease, PIMS-TS might be the result of thymic dysfunction caused by infection of the thymus with SARS-CoV-2, leading to delayed disease, reminiscent of severe autoimmunity. We found that human TEC expresses ACE2, confirming a recent study [35]. We also showed that human thymus explants can be infected with SARS-CoV-2 *in vitro*, and propagate the virus at 48 hours after infection, suggesting that SARS-CoV-2 infection could in theory affect thymus function and T-cell development. However, as we were unable to test our hypothesis directly by examining the thymuses of paediatric PIMS-TS patients, we employed a bulk population-based strategy to investigate the TCRβ and TCRɑ chain repertoires of children hospitalized with PIMS-TS and compared this to age-matched healthy children and severe paediatric COVID-19 patients who were admitted to the same PICU during 2021 and 2022, to look for evidence that supported or discounted the hypothesis. Given that PIMS-TS is a rare condition, our patient groups were necessarily small [27]. Despite this, we were able to identify many statistically significant changes in the TCR repertoires of hospitalized paediatric PIMS-TS patients, which highlight the strong effect of PIMS-TS on the TCR repertoire.

TCR sequencing showed that while CD4 and CD8 T-cell populations were depleted in PBMC of both PIMS-TS and COVID-19 patient groups, the diversity and distribution of their TCRβ and TCRɑ repertoires were grossly normal. This is in contrast to TCRβ repertoires sequenced from adult COVID-19 patients, which showed increased clonality and reduction in diversity in severe disease [69]. The fact that children with severe COVID-19 displayed normal TCR repertoire diversity might reflect more active thymus function in children than adults, enabling efficient replenishment of the peripheral T-cell pool on depletion. In support of this, younger healthy children show higher proportions of naïve and RTE T cells in their blood than older healthy children [10], and we found no differences in the proportions of naïve or RTE cells in CD4 or CD8 populations from children with severe COVID-19 compared to healthy control children in the same age band. Interestingly, the CD4 population in the PIMS-TS group contained a greater proportion of naïve cells than healthy children, and we observed close positive correlations between the number of TREC in PBMC and the proportion of naive cells in the CD8 and CD4 populations in PIMS-TS patients, suggesting that in contrast to the healthy children, the naïve T-cell pool in PIMS-TS patients is made up largely of RTE, with little contribution from peripheral homeostatic proliferation.

Analysis of TCR gene segment usage revealed many distinctive features of the TCR repertoires of the PIMS-TS group, with enrichment of TCRβ sequences using TRBV11-2, TRBV12-4 and TRBV29-1, and increased proportional combinatorial usage of several combinations, such as TRBV11-2 × TRBJ2-7, TRBV11-2 × TRBJ1-1, TRBV11-2 × TRBJ2-5, TRBV11-2 × TRBJ2-1, TRBV29-1 × TRBJ2-7, TRBV29-1 × TRBJ1. Consistent with this, PCA of combinatorial V × J frequencies for the top 5% most abundant clonotypes in both TCRɑ and TCRβ repertoires separated PIMS-TS from healthy repertoires on PC1, demonstrating the different V × J combinatorial usage in their repertoires.

The TRBV11-2 gene segment was also found to be over-represented in TCR sequencing from PBMC from several paediatric cohorts of severe PIMS-TS/MIS-C patients in 2020 from the USA and Europe [28–31], demonstrating consistency of the disease’s impact on the TCR repertoire across different COVID-19 waves and geographical locations. The TRBV11-2 expansion in American PIMS-TS patients was associated with HLA-I alleles, and it was suggested that it was caused by a Superantigen-like motif in the spike protein driving clonal TRBV11-2 expansion, leading to hyperinflammation and toxic shock [29, 30, 32]. Interestingly, V × J combinations using TRBV11-2 and TRBV12-4 were also enriched in our non-productive PIMS-TS sequences, suggesting that their enrichment in PIMS-TS patients might in part be accounted for by differences in TCR gene rearrangement in the thymus of PIMS-TS patients, rather than being the result of selection of these clonotypes in response to the virus. In contrast, in the COVID-19 CD8 repertoires, combinations using TRBV27 and TRBV4-1 were enriched in comparison to healthy controls, but these combinations were not proportionally increased in the non-productive sequences.

Comparison of proportional combinatorial productive TCRɑ V × J usage also showed enrichment of several different combinations in the two disease groups. Interestingly, when we compared proportional combinatorial non-productive TCRɑ V × J usage, we found that the V × J combinations that were increased in the PIMS-TS repertoires compared to control were enriched for distal (5′ TRAV to 3′ TRAJ) rearrangements and that in general we detected more combinations of distal rearrangements than proximal rearrangements in the non-productive PIMS-TS sequences. This bias towards distal rearrangements in the non-productive sequences provides evidence for an impact of PIMS-TS on the process of TCRɑ gene rearrangement in the thymus, as these biases cannot have been selected through protein interactions. Distal TCRɑ rearrangements take place later in time after initiation of TCRɑ locus rearrangement in each cell than proximal rearrangements, and so are consistent with slower differentiation. This could occur if TEC numbers were reduced, because of TEC infection by SARS-CoV-2 leading to TEC cell death, as has been described in a recent study [35]. A reduction in TEC could mean that thymocytes were unable to bind MHC+peptide ligand on TEC for positive selection, and so would continue to rearrange along the TCRɑ loci, leading to enrichment of 5′ to 3′ TCRɑ rearrangements. Thymic emigrants that have not been adequately positively selected might fail to expand normally in a lymphopenic environment, as peripheral homeostatic expansion requires tonic TCR-MHC interactions [70]. This could explain the close correlation between the proportion of naïve T cells and TREC numbers in the PIMS-TS patients.

In contrast, although the COVID-19 group showed increased proportional usage of several different TRAV-TRAJ combinations, with enrichment of TRAV3-2 in the CD4 population, and TRAV21 in the CD8 population, we found no differences in proportional non-productive TRAV × TRAJ combinations between the COVID-19 and healthy control groups.

In summary, our study has identified distinctive characteristics of the TCR repertoires of children hospitalized with PIMS-TS in 2021 and 2022. We found several similar features to previously described repertoires from PIMS-TS 2020 cohorts, as well as novel features of their TCR repertoires [28–31]. Although our analyses do not definitively demonstrate thymus involvement in PIMS-TS, several aspects of these TCR repertoires are suggestive of thymus dysfunction, including changes in proportional combinatorial V × J usage in productive and non-productive TCRβ and TCRɑ sequences with bias to 5′TRAV × 3′TRAJ rearrangements. Differences in thymus function between healthy children and PIMS-TS could be the result of SARS-CoV-2 infection of the thymus in PIMS-TS children, or alternatively, PIMS-TS could be linked to a pre-existing abnormality in thymus function or T-cell intrinsic abnormality in T-cell development. Interestingly, candidate genes whose autosomal recessive deficiencies have been associated with PIMS-TS (*OAS1/2* and *RNASEL)* are expressed in TEC and developing T cells in the thymus.

We believe that it will be important to consider further the involvement of thymus dysfunction in PIMS-TS should more cases arise. Given the similarities between PIMS-TS and Kawasaki disease, it will also be interesting to investigate possible thymus dysfunction in this patient group.

## Supplementary Material

uxaf027_suppl_Supplementary_Figure_S1

uxaf027_suppl_Supplementary_Figure_S2

uxaf027_suppl_Supplementary_Figure_S3

uxaf027_suppl_Supplementary_Figure_S4

uxaf027_suppl_Supplementary_Figure_S5

uxaf027_suppl_Supplementary_Table_S1

uxaf027_suppl_Supplementary_Table_S2

uxaf027_suppl_Supplementary_Figure_Legends

## Data Availability

TCR sequencing data is available on UCL Research Data Repository at https://doi.org/10.5522/04/24807900.v1

## Abbreviation

COVID-19: coronavirus disease 2019
GOSH: Great Ormond Street Hospital
HRA: Health Research Authority
MIS-C: a multisystem inflammatory syndrome in children
PBMC: Peripheral blood mononuclear cells
PIMS-TS: paediatric inflammatory multisystem syndrome temporally associated with COVID-19
TEC: thymic epithelial cells
TCR: T-cell receptor
TRA: T-cell Receptor alpha locus
TRB: T-cell Receptor Beta locus
TSS: Toxic Shock Syndrome
TREC: T-cell receptor excision circles
