# Supplementary figures and images for: Distinctive T-cell receptor repertoire in paediatric inflammatory multisystem syndrome temporally associated with coronavirus disease 2019/multisystem inflammatory syndrome in children patients: possible thymus involvement
Source: Clin Exp Immunol. 2025 May 4;219(1):uxaf027. doi: 10.1093/cei/uxaf027 (PMC12202041; doi:10.1093/cei/uxaf027)

Supp Figure 1

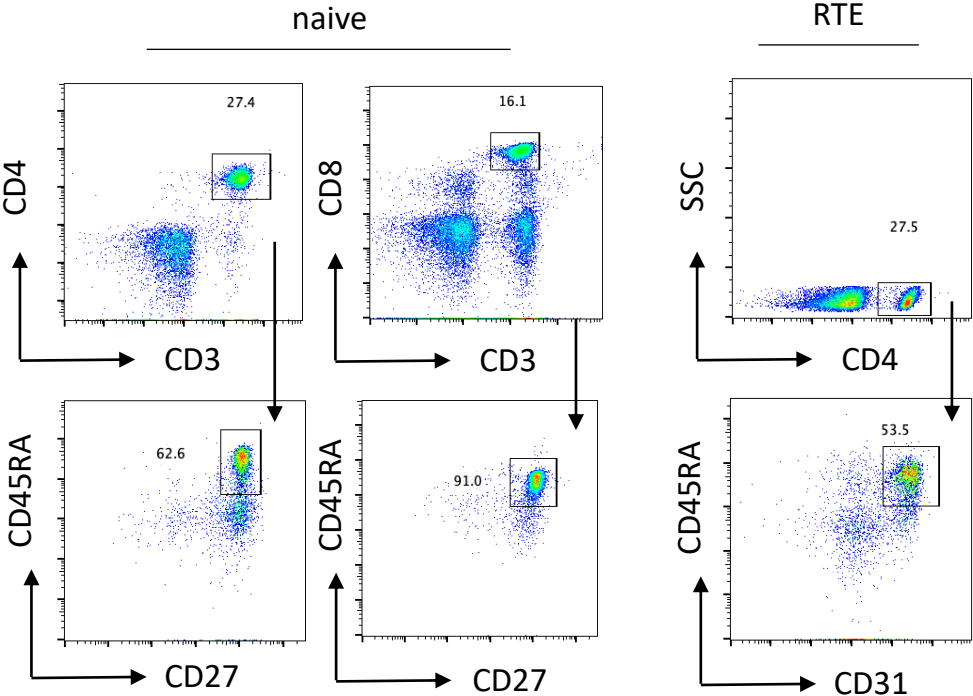

Supplement: uxaf027_suppl_Supplementary_Figure_S1 [file uxaf027_suppl_supplementary_figure_s1.pdf]

**A**

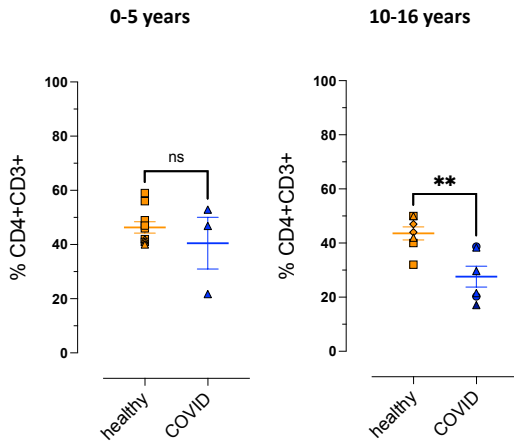

**B**

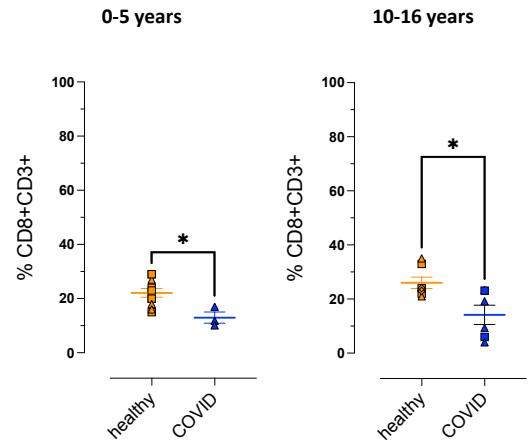

**C**

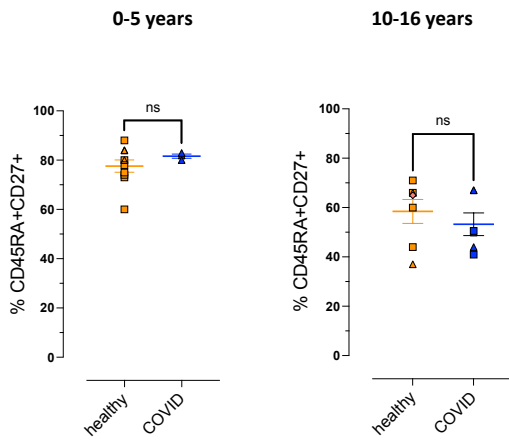

**D**

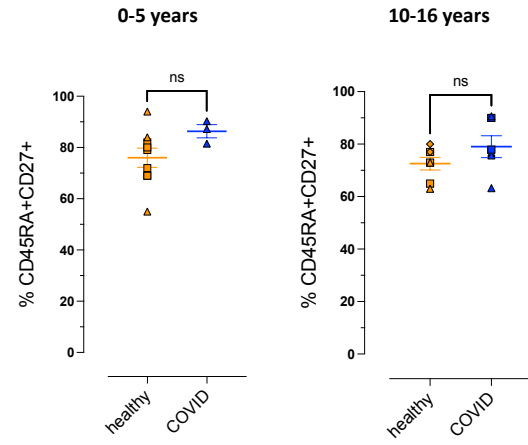

**E**

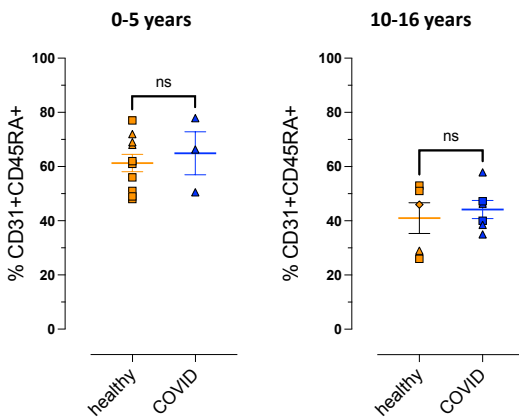

Supplement: uxaf027_suppl_Supplementary_Figure_S2 [file uxaf027_suppl_supplementary_figure_s2.pdf]

A

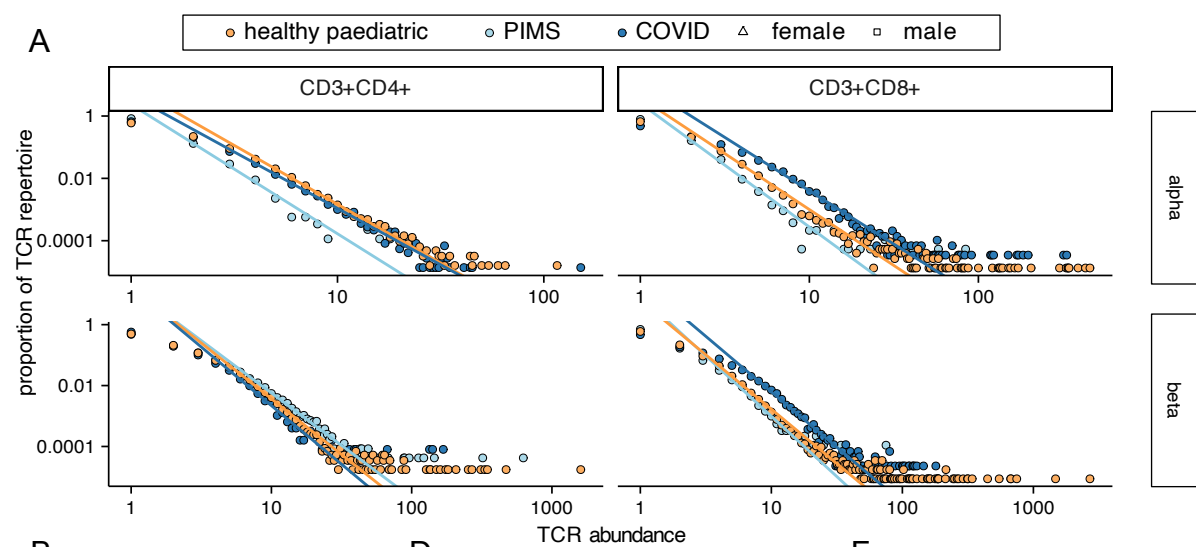

B

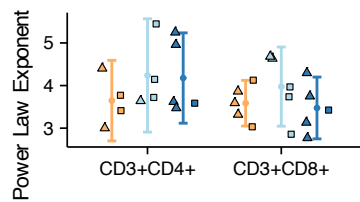

D

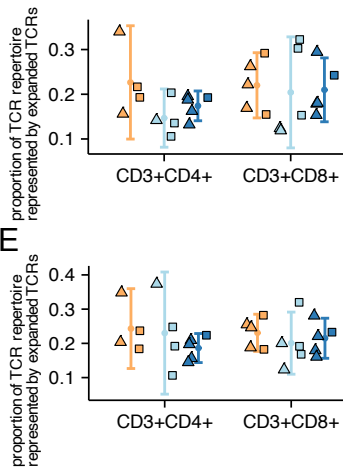

F

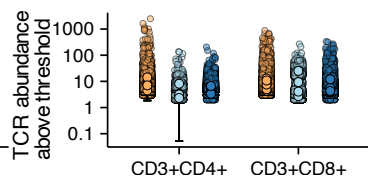

C

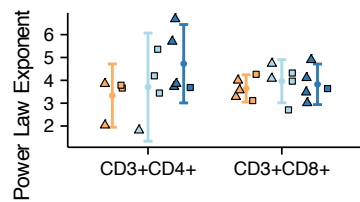

E

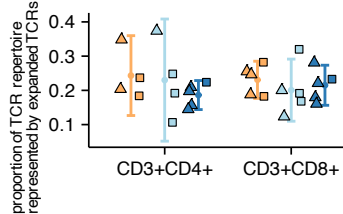

G

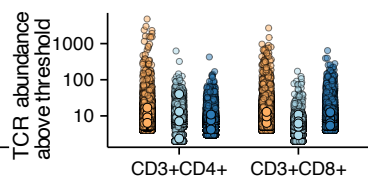

H

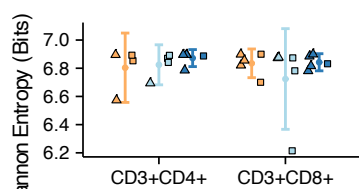

J

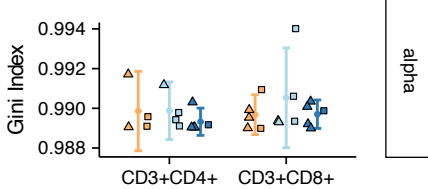

I

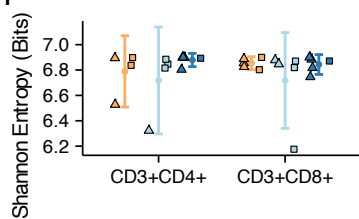

K

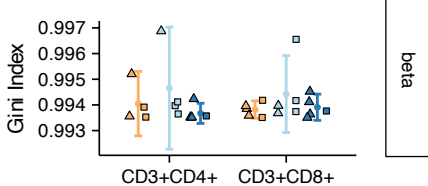

Supplement: uxaf027_suppl_Supplementary_Figure_S3 [file uxaf027_suppl_supplementary_figure_s3.pdf]

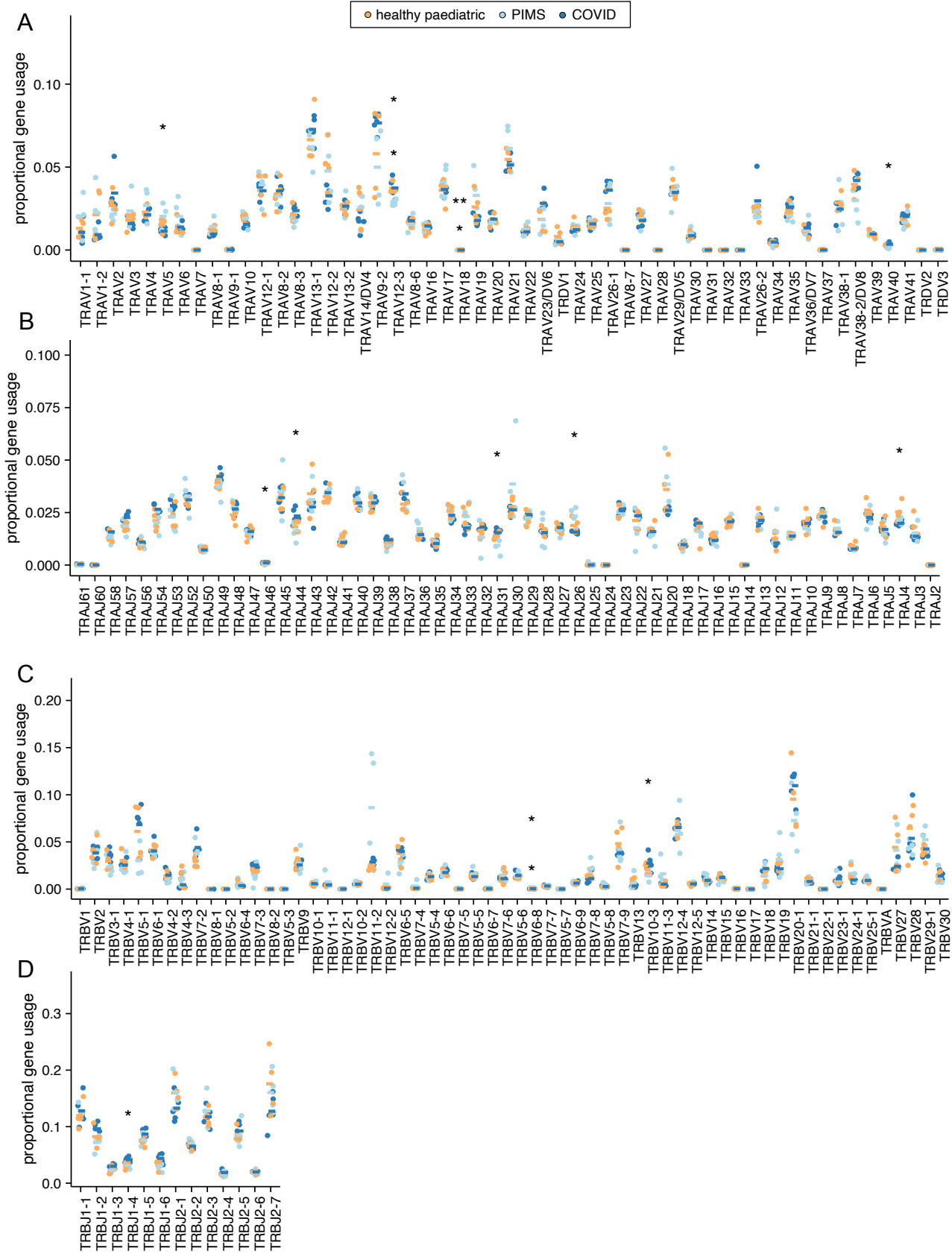

Supplement: uxaf027_suppl_Supplementary_Figure_S4 [file uxaf027_suppl_supplementary_figure_s4.pdf]

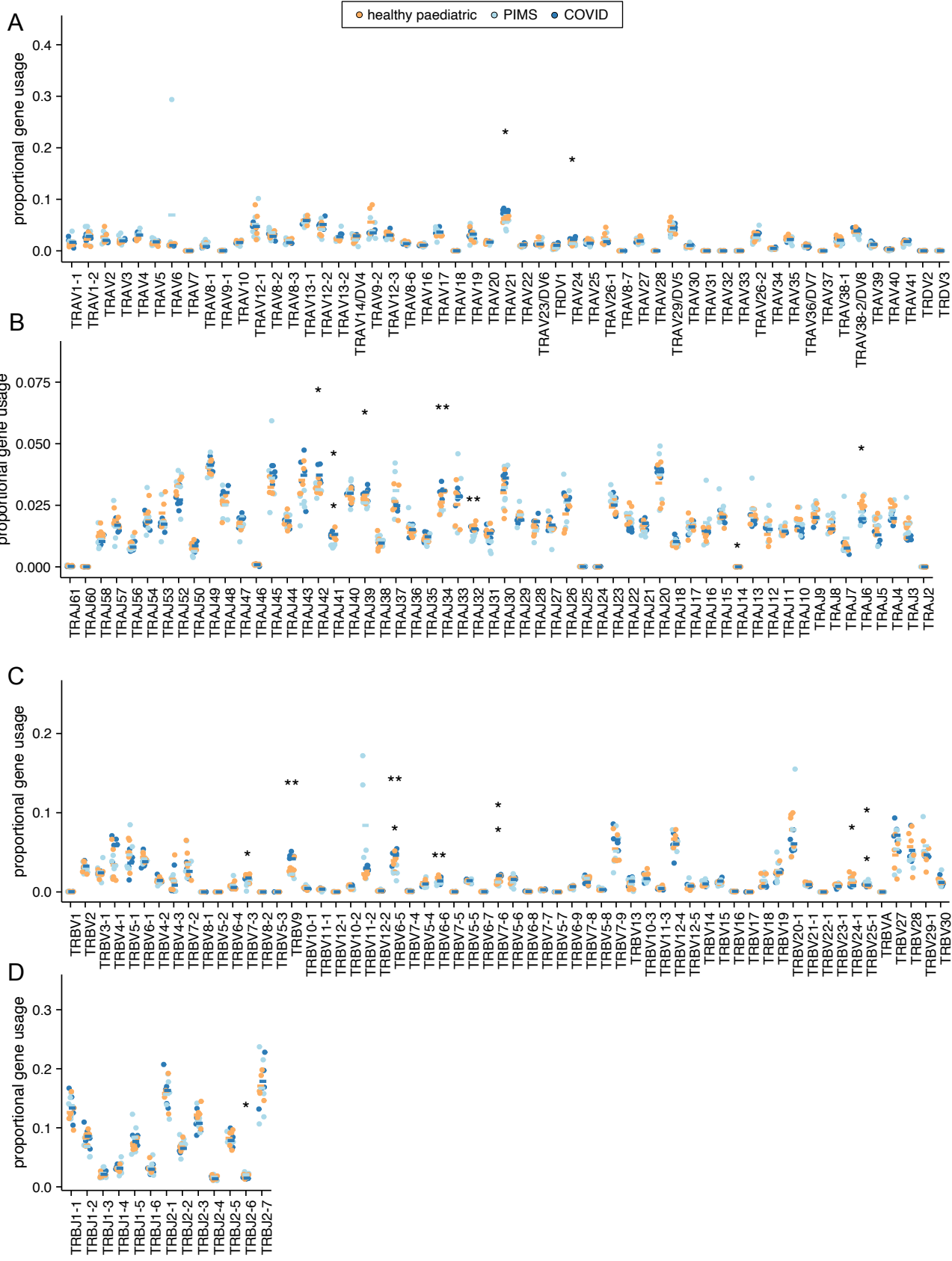

Supplement: uxaf027_suppl_Supplementary_Figure_S5 [file uxaf027_suppl_supplementary_figure_s5.pdf]
