# Supplementary material for: Distinctive T-cell receptor repertoire in paediatric inflammatory multisystem syndrome temporally associated with coronavirus disease 2019/multisystem inflammatory syndrome in children patients: possible thymus involvement
Source: Clin Exp Immunol. 2025 May 4;219(1):uxaf027. doi: 10.1093/cei/uxaf027 (PMC12202041; doi:10.1093/cei/uxaf027)
Supplement: uxaf027_suppl_Supplementary_Table_S1 [file uxaf027_suppl_supplementary_table_s1.docx]

**Supp Table 1. List of antibodies**

| **ANTIBODY** | **FLUOROCHROME** | **CLONE** |
| --- | --- | --- |
|  |  |  |
| CD4 | PerCP-Cyanine5.5 | RPA-T4 |
| CD4 | APC | RPA-T4 |
| CD8 | APC | RPA-T8 |
| CD3 | PerCP-Cyanine5.5 | OKT3 |
| CD31 | PE | WM59 |
| CD27 | PE | O323 |
| CD27 | APC | O323 |
| CD45RA | FITC | HI100 |
| DAPI |  |  |
