# Supplementary material for: Distinctive T-cell receptor repertoire in paediatric inflammatory multisystem syndrome temporally associated with coronavirus disease 2019/multisystem inflammatory syndrome in children patients: possible thymus involvement
Source: Clin Exp Immunol. 2025 May 4;219(1):uxaf027. doi: 10.1093/cei/uxaf027 (PMC12202041; doi:10.1093/cei/uxaf027)
Supplement: uxaf027_suppl_Supplementary_Figure_Legends [file uxaf027_suppl_supplementary_figure_legends.docx]

**Supplementary Figure legends**

**Supplementary Figure 1: Representative flow cytometry plots used to identify T-cell populations from PBMCs**

Plots show representative flow cytometry that was used to identify T-cell populations from PBMC shown in Fig2 and SFig2.

**Supplementary Figure 2: Lymphocyte populations in blood of paediatric age-matched healthy control and COVID-19 patients.**

**(A-E)** Percentages of T-cells, naïve T-cells and CD4 RTE in PBMC prepared from blood from paediatric healthy control (healthy, orange) and COVID-19 (COVID, dark blue) patients in age ranges of <5 years old and 10-16 years old, determined by flow cytometry. (**A**) CD4+CD3+ (**B**) CD8+CD3+. (**C-E**) percentages are gated on CD4+CD3+ (**C-D**) and CD8+CD3+ (**E**): CD45RA+CD27+ (naïve CD4); (**D**): CD45RA+CD31+ (CD4 RTE); (**E**) CD45RA+CD27+ (naïve CD8). Each dot represents an individual child. Plots show mean + SEM.; **p <*0.05 ** *p <*0.01 unpaired Student’s t-test.

**Supplementary Figure 3: Comparison of TCR diversity and clonality between healthy paediatric, PIMS and COVID-19 groups**

TCR α- and β-chain repertoires were sequenced from FACS-sorted CD3+CD4+ and CD3+CD8+ populations from PBMC isolates from healthy children (healthy paediatric, orange circles; n=4/5), PIMS-TS patients (PIMS, light blue circles, n=4/5) and COVID-19 patients (COVID, dark blue circles; n=5) in 10-16 year age range.

**(A)** The frequency distribution of TCR α (upper) and β-chain (lower) abundance was fitted to
a discrete power law (*f*(*k*) = *Ck*^−^*^α^*) by maximum likelihood (solid line). These logged plots show a representative plot of an individual patient for healthy paediatric, PIMS-TS and COVID-19 groups in CD3+CD4+ and CD3+CD8+ populations. The *x* axis represents TCR abundance (size of clonotype), and the *y* axis represents the proportion of the repertoire. The negative of the power law exponent corresponds to the slope of the logged plot. **(B, C)** The power law exponents of the frequency distribution of TCR α **(B)** and β-chain **(C)** abundances for healthy, PIMS-TS and COVID-19 groups in CD3+CD4+ and CD3+CD8+ populations, where each point represents an individual. **(D, E)** The proportion of the total TCR α **(D)** and β-chain **(E)** repertoire accounted for by the expanded top 5% most abundant sequences (>99^th^ percentile) for healthy, PIMS and COVID groups in CD3+CD4+ and CD3+CD8+ populations, where each point represents an individual. **(F, G)** The number of TCR α **(F)** and β-chain **(G)** sequences detected above the given frequency threshold (top 5% most abundant sequences) is shown for healthy controls, PIMS-TS and COVID-19 groups in CD3+CD4+ and CD3+CD8+ populations. Each small translucent point represents the abundance of any α- or β-chain sequence detected above the threshold for all patients in that group, while each larger solid point represents the mean of α- or β-chain sequence abundance for each individual. **(H-I)** Index of Shannon entropy for TCRα (**H**) and TCRβ (**I**) repertoires from the three groups. (**J-K**) Gini index for TCRα (**H)** and TCRβ (**I**) repertoires from the three groups.

(**B-E** and **H-K**) In dot plots each dot represents a different individual. Dot plots show mean±c.i.

Statistical comparisons were carried out by one-way ANOVA followed by Tukey post-hoc tests.

**Supplementary Figure 4: PIMS-TS and COVID-19 patients’ CD3+CD4+ TCR repertoires favour distinct V and J gene segments.**

TCR α-chain and β-chain repertoires were sequenced from FACS-sorted CD3+CD4+ populations from PBMCs isolated from healthy paediatric controls (healthy paediatric, orange circles; n=4/5), PIMS-TS patients (PIMS, light blue circles, n=4/5) and COVID-19 patients (COVID, dark blue circles; n=5) in 10-16 year age range.

**(A-D)**Dotplots show proportional TRAV (**A**), TRAJ (**B**), TRBV (**C**), and TRBJ (**D**) gene usage of total TCRs for healthy paediatric controls (healthy paediatric, orange circles; n=4/5), PIMS-TS patients (PIMS, light blue circles, n=4/5) and COVID-19 patients (COVID, dark blue circles; n=5) in CD3+CD4+ populations. Genes are shown in chromosomal order (5’ to 3’) from left to right. Dots represent individual patients and bars show the mean. Statistical comparisons were carried out by one-way ANOVA followed by Tukey post-hoc tests (significant *p*values shown):** *p <*0.01; **p*< 0.05.

**Supplementary Figure 5: PIMS-TS and COVID-19 patients’ CD3+CD8+ TCR repertoires favour distinct V and J gene segments.**

TCR α-chain and β-chain repertoires were sequenced from FACS-sorted CD3+CD8+ populations from PBMCs isolated from healthy paediatric controls (healthy paediatric, orange circles; n=4/5), PIMS-TS patients (PIMS, light blue circles, n=4/5) and COVID-19 patients (COVID, dark blue circles; n=5) in 10-16 year age range.

**(A-D)**Dotplots show proportional TRAV (**A**), TRAJ (**B**), TRBV (**C**), and TRBJ (**D**) gene usage of total TCRs for healthy paediatric controls (healthy paediatric, orange circles; n=4/5), PIMS-TS patients (PIMS, light blue circles, n=4/5) and COVID-19 patients (COVID, dark blue circles; n=5) in CD3+CD4+ populations. Genes are shown in chromosomal order (5’ to 3’) from left to right. Dots represent individual patients and bars show the mean. Statistical comparisons were carried out by one-way ANOVA followed by Tukey post-hoc tests (significant *p*values shown): ** *p <*0.01; **p*< 0.05.

**Supplementary Tables**

**Supplementary Table 1**

Antibodies used for flow cytometry

**Supplementary Table 2**

PCR conditions used for analysis of SARS-CoV-2 subgenomic RNA
