# Supplementary material for: Distinctive T-cell receptor repertoire in paediatric inflammatory multisystem syndrome temporally associated with coronavirus disease 2019/multisystem inflammatory syndrome in children patients: possible thymus involvement
Source: Clin Exp Immunol. 2025 May 4;219(1):uxaf027. doi: 10.1093/cei/uxaf027 (PMC12202041; doi:10.1093/cei/uxaf027)
Supplement: Supplementary file 4 [file uxaf027_suppl_supplementary_table_s2.docx]

Supplementary Table 2 RT-PCR program for *E* and *N2* genes

Envelope (E)		
**STEP**	**TIME**	**TEMPERATURE**
Initial PCR activation step	30s	95°C
Denaturation	5s	95°C
Annealing	15s	60°C
Extension	35s	72°C
Number of cycles	45 cycles	
Nucleocapsid 2 (N2)		
**STEP**	**TIME**	**TEMPERATURE**
Initial PCR activation step	5 min	95°C
Denaturation	15s	95°C
Annealing	1min	60°C
Extension	30s	68°C
Final extension	5min	68°C
